# The Influence of Energetic Factors on Biomarkers of Postmenopausal Breast Cancer Risk

**DOI:** 10.1007/s13668-013-0069-8

**Published:** 2013-12-15

**Authors:** Heather K. Neilson, Shannon M. Conroy, Christine M. Friedenreich

**Affiliations:** 1 Department of Population Health Research, CancerControl Alberta, Alberta Health Services, Quarry Park, c/o 10101 Southport Rd SW, Calgary, Alberta T2W 3N2 Canada; 2 Department of Community Health Sciences, Faculty of Medicine, University of Calgary, 3330 Hospital Drive N.W., Calgary, Alberta T2N 4N2 Canada; 3 Department of Oncology, Faculty of Medicine, University of Calgary, 1331 29 St. N.W., Calgary, Alberta T2N 4N2 Canada

**Keywords:** Breast cancer, Postmenopausal women, Randomized trials, Physical activity, Exercise, Weight loss, Adiposity, Biomarkers, Sex hormones, Estrogen, Biomechanisms

## Abstract

Strong and consistent evidence exists that physical activity reduces breast cancer risk by 10-25 %, and several proposed biologic mechanisms have now been investigated in randomized, controlled, exercise intervention trials. Leading hypothesized mechanisms relating to postmenopausal breast cancer include adiposity, endogenous sex hormones, insulin resistance, and chronic low-grade inflammation. In addition, other pathways are emerging as potentially important, including those involving oxidative stress and telomere length, global DNA hypomethylation, immune function, and vitamin D exposure. Recent exercise trials in overweight/obese postmenopausal women implicate weight loss as a mechanism whereby exercise induces favorable changes in circulating estradiol levels and other biomarkers as well. Still it is plausible that some exercise-induced biomarker changes do not require loss of body fat, whereas others depend on abdominal fat loss. We highlight the latest findings from randomized, controlled trials of healthy postmenopausal women, relating exercise to proposed biomarkers for postmenopausal breast cancer risk.

## Introduction

There is convincing epidemiologic evidence that body fatness, and probable evidence that adult weight gain, are associated with an increased risk of postmenopausal breast cancer [1]. One meta-analysis demonstrated that every 5 kg/m^2^ increase in body mass index (BMI) increases postmenopausal breast cancer risk by 12 % on average [2] with possible variation by tumor subtype [3]. In addition, after a breast cancer diagnosis, survival rates are decreased with higher BMI [4] by as much as 30 % [5].

It is of great interest, therefore, to prospectively study the effects of weight control on breast cancer risk and survival in overweight or obese postmenopausal women [6]. Yet first, understanding the contributions of energetic factors—i.e., physical activity and diet—is needed to determine the optimal weight control intervention. Regular physical activity is a widely accepted health-promoting behavior that is recommended for cancer prevention [1, 7], but the *type* and *dose* of activity that is optimal for postmenopausal breast cancer prevention remains unclear.

### Physical activity and breast cancer

Convincing epidemiologic evidence suggests that physical activity of moderate-to-vigorous intensity reduces breast cancer risk by 10-25 % on average relative to inactivity [8•, 9] The dose of activity required for breast cancer prevention is unclear, but across observational studies, risk generally decreases with higher physical activity duration [8•] and intensity [9]. For cancer prevention overall, public health recommendations advise at least 30 minutes of moderate-intensity activity equivalent to brisk walking every day [1] or 30 minutes or more of moderate-to-vigorous activity at least 5 days per week [7] for adults.

### Biomarker studies in healthy women

Given clear inverse relations between physical activity and future postmenopausal breast cancer risk, lifestyle modification for inactive women holds promise for breast cancer prevention. Whereas randomized, controlled trials (RCTs) examining breast cancer outcomes would best inform an exercise prescription, these trials have not been conducted because of the large sample size and time required for a prospective study. A more feasible approach is to study the impact of lifestyle change on breast cancer biomarkers using a RCT [10]. The number of exercise RCTs studying proposed biomarkers for breast cancer has escalated during the past 10 years, shedding light on: 1) exercise prescriptions that might impact breast cancer risk, and 2) underlying biologic mechanisms.

We provide an update on the epidemiologic evidence relating exercise to proposed biomarkers for postmenopausal breast cancer risk, without dietary modification. This review enhances our earlier reviews [8, 11] by focusing on the strongest, most up-to-date epidemiologic evidence (from randomized trials) relating exercise to estrogens and adiposity, the two most convincing biomarkers of postmenopausal breast cancer risk. We also update our biologic model that relates physical activity to breast cancer by incorporating newly hypothesized biomarkers. Our goal is to guide future clinical and mechanistic research surrounding physical activity and postmenopausal breast cancer prevention. The focus of this review will be on healthy women in whom biomarker profiles and the types and effects of prescribed exercise may differ from breast cancer survivors. Biomarker studies in breast cancer survivors are reviewed elsewhere [12•, 13].

## Epidemiologic evidence relating exercise to proposed biomarkers

To simplify our discussion of the existing epidemiologic evidence, we have classified proposed biomarkers of breast cancer risk as “convincing” or “hypothesized.” Our classification of estrogens and adiposity as convincing biomarkers is based on the relatively strong and consistent body of epidemiologic evidence relating these markers to postmenopausal breast cancer risk. We summarize that evidence and describe in more detail the effects of exercise on estrogen levels (by systematically reviewing RCT evidence) and adiposity (citing recent reviews and large RCTs), specifically in postmenopausal women. We then provide a high-level overview of other, hypothesized biomarkers of postmenopausal breast cancer risk and possible relations with exercise.

### Systematic review of RCTs relating exercise to estrogens

In September 2013, we searched the published literature (PubMed-NIH) for RCTs that studied the impact of exercise on estrogens. In brief, we identified all RCTs of long-term exercise (≥4 weeks) that compared exercise-only to a nonexercise control group in healthy postmenopausal women. Studies exclusive to hormone replacement therapy users were excluded as were studies in morbidly obese women (BMI >40 kg/m^2^) and trained athletes.

### Convincing biomarkers for postmenopausal breast cancer risk

#### Adiposity

Body fatness is an accepted, convincing biomarker for increased postmenopausal breast cancer risk in healthy women [1, 2]. Multiple interrelated biologic pathways could mediate the association between adiposity and postmenopausal breast cancer, with sex hormones, insulin resistance, and low-grade chronic inflammation as leading hypotheses [14•, 15]. Furthermore, central adiposity may be particularly important. Recently in postmenopausal women, independently of BMI, waist circumference was positively associated with breast cancer risk [16] and abdominal fat was related to sex hormone bioavailability [17•], which is a strong biomarker of breast cancer risk. Therefore, with respect to postmenopausal breast cancer, there may be more benefit from exercise prescriptions that can effectively lower abdominal fat.

Exercise is publicly recommended for modest weight loss, for prevention of weight gain in overweight and obese adults, and for prevention of weight regain after weight loss [18, 19]. Exercise trials typically produce <3 % weight loss in adults, although more might be achieved with higher volumes of exercise, e.g., the American College of Sports Medicine recently proposed >250 minutes per week at moderate intensity [18]. However exercise-induced weight loss could vary by age. In a prospective, observational study of 58,610 postmenopausal women, whether body weight was lost, maintained, or gained with high levels of physical activity generally depended on the age of the women at baseline [20]. Also, exercise type could be relevant, e.g., aerobic may be preferable to resistance exercise with respect to weight loss [18] and lowering total abdominal fat [21•] in overweight adults. Indeed, the largest exercise RCTs of moderate-vigorous aerobic exercise in healthy postmenopausal women all showed decreases in intra-abdominal fat [22, 23] or waist circumference [24•, 25, 26] and overall body fat [22, 23, 24•, 25].

#### Sex hormones

Higher levels of endogenous estrogens and androgens and lower levels of circulating sex hormone binding globulin (SHBG) are related to an increased risk of postmenopausal breast cancer [27]. In addition, associations between postmenopausal breast cancer risk and hormone replacement therapy use [28] and effective use of antiestrogenic drugs to prevent breast cancer [29] firmly support a causal role for estrogens. Estrogens can decrease apoptosis and act as mitogens in the breast via estrogen receptor binding; moreover, oxidative estrogen metabolites act as mutagenic, genotoxic agents possibly contributing to breast cancer initiation [30].

A total of nine exercise-only RCTs in postmenopausal women that studied changes in estrogen-related biomarkers for breast cancer were identified in our systematic review of published literature (Table 1). The number of non-HRT users assigned to exercise-only or control groups ranged from 16 [31] to 320 [32]. All study populations were overweight or obese on average, and all but one RCT of women 65+ years [33] studied younger postmenopausal women with mean ages from 54–61 years. Roughly half of the trials, comprising the four largest RCTs [32, 34, 35, 36••] and one smaller RCT [37], involved 12-month interventions, whereas the remainder were 12 [31, 33] or 16 [38, 39] weeks duration. Most interventions were aerobic [32, 33, 35, 36••, 38] or combined aerobic/resistance training [34, 37]; two small RCTs focused on resistance training [31, 39]. Exercise prescriptions ranged from 150–225 minutes/week (except [31] where minutes per week were not reported) and were generally moderate-vigorous intensity (i.e., 60-85 % maximum heart rate).

**Table 1:** Summary of randomized controlled trials of long-term exercise that studied estrogen changes in cancer-free, postmenopausal women

Trial name/reference, country	Sample size^a^	Study participants	Intervention arm prescription	Comparison group(s)
Figueroa et al., 2003, USA [37]	n = 24 EX; n = 28 CTL	• Mean body fat, 39 %; mean body weight, 67–71 kg • Inactive • No HRT use (HRT users analyzed separately) • Age 40–65 yr; mean, 57 yr	• 12 mo • 60–75 min/day, 3 days/wk, supervised • Resistance and weight-bearing aerobic exercise • 7 resistance exercises, 2 sets @ 70-80 % 1-RM + 25 min aerobic exercise @ 50-80 % HR_max_	Maintained usual level of physical activity
Copeland et al., 2004, Canada [31]	n = 8 EX; n = 8 CTL	• Mean BMI 26 kg/m^2^ (EX); 32 kg/m^2^ (CTL) • No regular exercise in past year • No HRT use (HRT users analyzed separately) • Mean age, 54 yr	• 12 wk • 3 days/wk, supervised • Resistance training • By 1 month, progressed to 8 exercises @ 3 sets,10 repetitions each	Flexibility exercises 3 days/wk, unsupervised
Physical Activity for Total Health Study, USA [35]	n = 87 EX; n = 86 CTL	• BMI 25–40 kg/m^2^, mean 30 kg/m^2^; body fat >33 % • Previously <60 min/week exercise that caused sweating • No hormone use past 6 mo • Age 50–75 yr; mean, 61 yr • 86 % non-Hispanic white	• 12 mo • 45 min/day, 5 days/wk (supervised and home-based) • Aerobic exercise • 60-75 % HR_max_ by wk 8	Stretching controls
Orsatti et al., 2008, Brazil [39]	n = 22 EX; n = 21 CTL	• Mean BMI 28–29 kg/m^2^, mean body fat 33-36 % • No previous leisure activity besides household • No hormone therapy past 6 mo • Age 40–70 yr; mean, 58–59 yr	• 16 wk preceded by 4-wk low-load adaptation period • 50–60 min/day, 3 days/week, supervised • Resistance training • 8 exercises @ 3 sets, 8–12 repetitions each, 60-80 % 1-RM	Asked not to change exercise habits
Sex Hormones and Physical Exercise (SHAPE) study, the Netherlands [34]	n = 96 EX; n = 93 CTL	• BMI 22–40 kg/m^2^, mean 27 kg/m^2^; mean body fat 40-41 % • <2 hr/wk moderate sport/recreational activity and not adherent to international physical activity recommendations • No HRT use past 6 mo • Age 50–69 yr; mean, 58–59 yr	• 12 mo • 60 min/day, 2 days/wk supervised group session + 30 min/week home-based individual session • Supervised sessions: aerobic (20 min @ 60-85 % HR_max_) and strength training (25 min) + warm-up, cool-down • Home-based sessions: brisk walking or cycling @ 60-80 % HR_max_ (30 min)	Asked to retain habitual exercise patterns
Alberta Physical Activity and Breast Cancer Prevention (ALPHA) trial, Canada [32]	n = 160 EX; n = 160 CTL	• BMI 22–40 kg/m^2^, mean 29 kg/m^2^ • <90 min/wk recreational activity or if between 90–120 min/wk had maximal oxygen uptake <34.5 mL/kg/min • No hormone use • Age 50–74 yr; mean, 61 yr • 91 % white race	• 12 mo • 45 min/day, 5 days/wk (supervised and home-based) • Aerobic exercise, mainly walking or cycling • At least half of each workout @ 70-80 % heart rate reserve; achieved by wk 12	Maintained usual level of activity
Yoo et al., 2010, South Korea [33]	n = 11 EX; n = 10 CTL	• Mean BMI, 25–27 kg/m^2^ • No hormone use • Age >65 yr; mean age 71 yr	• 12 wk • 60 min/day, 3 days/wk, supervised • 45-min walking with two 1-kg ankle weights; 10-min warm-up + 5-min cool-down	Asked to maintain usual physical activity routine
Kim et al., 2012, South Korea [38]	n = 15 EX; n = 15 CTL	• Mean BMI, 25 kg/m^2^; >32 % body fat, mean 36 % • <20 min exercise twice weekly • No hormone use • Mean age 54 yr	• 16 wk • 60 min/day, 3 days/wk, supervised • Aerobic exercise • Line dancing, attained 70-80 % HR_max_ by wk 12	No exercise
Nutrition and Exercise for Women (NEW) Trial, USA [36••]	n = 117 EX; n = 87 CTL; n = 118 DIET; n = 117 DIET + EX	• BMI >25.0 kg/m^2^, mean 30.9 kg/m^2^, mean body fat 47.2 % • Moderate-intensity physical activity <100 min/week • No hormone use past 3 months • Age 50–75 yr; mean, 58 yr • 85 % non-Hispanic white	• 12 mo • ≥ 45 min/day, 5 days/wk (3 supervised and 2 home-based) • Aerobic exercise with metabolic equivalent ≥ 4 •70-85 % HR_max_ for 45 min by wk 7	1)Reduced-calorie weight loss diet 2)Combined reduced-calorie weight loss diet + aerobic exercise 3)Requested not to change exercise or dietary habits

EX, exercise group; CTL, control group

^a^Sample size at baseline, as reported for estrogen analysis [32, 34, 35, 36••]

Table 2 summarizes results for estradiol, the most biologically potent estrogen [15], and also for estrone and SHBG from RCTs in Table 1. Findings on other estrogen-related biomarkers are described below. Nearly all of the reports in our systematic review described circulating hormone levels [31–35, 36••, 37–39] with single articles describing analysis of urine [40] and adipose tissue [41]. Results across RCTs were remarkably consistent, generally showing average decreases in sex hormones and increases in SHBG levels in exercise groups, typically <10 % in magnitude. Yet only a few primary analyses demonstrated *statistically significant* differences between exercise and control groups with respect to change in total estradiol [32], free estradiol [32, 35], estrone [35, 36••], or SHBG [32, 38]. No statistically significant group differences were found in primary analyses of testosterone or androstenedione [31–34, 36••, 37, 39, 42]. In the SHAPE trial, however, a significant intervention effect was found for testosterone and androstenedione (decreased levels in exercisers versus controls) for the subgroup who lost >2 % body fat [34]. The Physical Activity for Total Health study [40] showed no significant differences after 12 months between exercise (n = 87) and control groups (n = 86) for changes in 2-hydroxyestrone, 16α-hydroxyestrone, or their ratio in urine. In an ancillary study of the NEW trial (n = 45) [41], subcutaneous adipose tissue was analyzed for expression of 82 candidate genes related to adipokines, proinflammatory cytokines, and sex hormones. Combining women from all four trial arms, greater weight loss after 6 months was associated with decreased gene expression related to estrogen biosynthesis, e.g., 17β-hydroxysteriod dehydrogenase 1, which converts estrone to estradiol.

**Table 2:** Evidence from randomized controlled trials relating exercise to estrogens in healthy postmenopausal women^a^

Proposed biomarker	Average biomarker change for exercise-only group^b^	Evidence of adiposity change as a potential mediator of sex hormone change	Study reference
Circulating estradiol	−9.5%^c^; NS at 13 wk	-----	[31]
	−7.7 %; NS at 3 mo −4.4 %; NS at 12 mo	Stronger decreases in exercisers who lost 0.5 % + body fat	[35]
	No change at 12 mo	-----	[37]
	−8 %; NS over 12 mo	−11.7 % at 12 mo in exercise group who lost >2 % body fat Change in estradiol was significantly associated with change in % body fat	[34]
	−12 % at 12 mo; *p* = 0.004 over 12 mo	Exercise effect was slightly attenuated but remained significant after statistical adjustment for body weight change Statistical tests for mediation implied mediation by change in % body fat or total body fat, but not intra-abdominal fat	[32, 54•]
	+20.5 % ^c^; NS at 3 mo	-----	[33]
	−4.9 %, NS at 12 mo	The decrease in the DIET + EX group (−20.3 %) was significantly greater than for the EX group; *p* < 0.001 Significantly greater differences between change in EX versus CTL across subgroups of increasing weight loss (*p*-trend < 0.01)	[36••]
Circulating free estradiol	−8.2 %, *p* = 0.02 at 3 mo −6.1 %; NS at 12 mo	Stronger decreases in exercisers who lost 0.5 % + body fat	[35]
	+7.9 % ^c^; NS at 16 wk	-----	[39]
	−7.3 %; NS over 12 mo	−11.4 % at 12 mo in exercise group who lost >2 % body fat Change in free estradiol was significantly associated with change in %body fat	[34]
	−12.9 % at 12 mo; *p* = 0.001 over 12 mo	Exercise effect was slightly attenuated but remained significant after statistical adjustment for body weight change Statistical tests for mediation implied mediation by change in % body fat, total body fat, intra-abdominal fat	[32, 54•]
	−4.7 %, NS at 12 mo	The decrease in the DIET + EX group (−26 %) was significantly different than for the EX group; *p* < 0.001 Significantly greater differences between change in EX versus CTL across subgroups of increasing weight loss (*p*-trend = 0.001)	[36••]
Circulating estrone	−3.8 %, *p* = 0.03 at 3 mo −1.8 %; NS at 12 mo	Stronger decreases in exercisers who lost 0.5 % + body fat	[35]
	No change at 12 mo	-----	[37]
	−9.7 %; NS over 12 mo	−23.7 % at 12 mo in exercise group who lost >2 % body fat Change in estrone was significantly associated with change in % body fat	[34]
	−5.4 %; NS over 12 mo	Remained NS after statistical adjustment for body weight change	[32]
	−5.5 %, *p* = 0.01 at 12 mo	The decrease in the DIET + EX group (−11.1 %) was significantly greater than for the EX group; *p* = 0.01 Significantly greater differences between change in EX versus CTL across subgroups of increasing weight loss (*p*-trend < 0.01)	[36••]
Circulating sex hormone binding globulin (SHBG)^d^	+5.7 %; NS at 3 mo +8.8 %; NS at 12 mo	Stronger increases in exercisers who lost 0.5 % + body fat	[35]
	−0.7 %; NS over 12 mo	+2.0 % at 12 mo in exercise group who lost >2 % body fat	[34]
	+3.2 % at 12 months; *p* = 0.001 over 12 mo	Effect of exercise was no longer statistically different from controls after statistical adjustment for body weight change Statistical tests for mediation implied mediation by % body fat, total body fat, intra-abdominal fat	[32, 54•]
	+6.2%^c^; *p* < 0.001 at 16 wk	-----	[38]
	−0.7 %, NS at 12 mo	The change in the DIET + EX group (+25.8 %) was significantly different than for the EX group; *p* < 0.001 Greater difference between change in EX versus CTL groups with more weight loss, but *p*-trend NS	[36••]

^a^RCTs of long-term exercise-only interventions with results exclusively for healthy postmenopausal women

^b^NS indicates a nonstatistically significant difference between the change in the exercise group versus the control group

^c^Percent change in the exercise group was not reported in the article and therefore was approximated from published results, as follows:

[average sex hormone level at follow-up – average sex hormone level at baseline] / [average sex hormone level at baseline] x 100 %

^d^SHBG binding decreases estradiol and testosterone bioavailability

A mediating role for weight loss in the causal pathway between exercise and decreased estrogen levels is plausible given that adipose tissue is the primary source of endogenous estrogens post-menopause [15]. Evidence of mediation by adiposity change, described in Table 2, generally supports this hypothesis. Moreover, in the NEW trial, substantially greater decreases in total and free estradiol levels occurred, on average, within the diet + exercise arm than with exercise-only (−20.3 % vs. −4.9 % for estradiol; −26 % vs. −4.7 % for free estradiol) [36••]. In a smaller weight loss trial of obese postmenopausal women, a 14 % average decrease in total fat mass was associated with a 24 % decrease in estradiol levels in breast ductal fluid [43•]. In a recent prospective study, a 12.7 % decrease in total estradiol was estimated for every 1 kg/m^2^ decrease in BMI in 84 postmenopausal women who lost weight [44]. Interestingly in another recent study of 1,180 postmenopausal women [17•], waist circumference and waist-to-hip ratio—independently of BMI—were associated with circulating SHBG, free estradiol, and free testosterone levels, implicating abdominal fat as a specific target for breast cancer prevention.

### Hypothesized biomarkers of postmenopausal breast cancer risk

#### Insulin resistance

Insulin resistance, characterized by hyperinsulinemia, is a major predictor of diabetes risk and of possible etiologic importance in breast cancer [45, 46]. Insulin receptor binding promotes mitosis and antiapoptotic effects in breast cancer cells, and also tumor cell migration and tumor-associated angiogenesis [47]. In addition, chronically elevated insulin can enhance estrogen bioactivity and promote activities of breast cancer-related adipokines [47] and IGF-1 [14•]. Metformin, a pharmacologic agent that improves insulin sensitivity, is undergoing clinical testing for improved breast cancer survival [14•, 48].

In the United States, at least 150 minutes per week of moderate-vigorous aerobic and resistance exercise is recommended for diabetes prevention in prediabetics [49]. In larger RCTs of postmenopausal women, long-term aerobic exercise groups experienced average decreases of approximately −4 % to −10.3 % in insulin [50, 51, 52•, 53], −2 % to −11.4 % in homeostatic model of assessment for insulin resistance (HOMA-IR) [50, 51, 52•], −3.7 % in C-peptide [52•], and a small decrease [53] or negligible change in circulating glucose [50, 51, 52•]. However, in the NEW trial [52•], HOMA-IR decreased an average of −24 % and −26 % in the diet and diet + exercise intervention groups, respectively, after 12 months, suggesting strongly that improved whole-body insulin sensitivity in the exercise-only group (−8.6 % decrease in HOMA-IR) resulted from weight loss and not exercise *per se*. Secondary analyses of ALPHA trial data [54•] implied partial mediation by change in intra-abdominal fat area, which is of etiologic relevance to insulin resistance [55], and by total (%) body fat but also mediation by other unidentified factors as well.

Changes in insulin sensitivity could vary with different exercise prescriptions. In large RCTs of postmenopausal women, the greatest improvement in HOMA-IR was found with aerobic exercise >225 minutes/week [50] and >130 minutes/week [51]. In the NEW trial, 225 minutes/week of prescribed aerobic exercise was associated with a regression to normal fasting glucose levels for those with impaired glucose tolerance at baseline [52•]. Resistance training could provide distinct benefits for glycemic control by altering the quality and quantity of skeletal muscle [56]. However, in an RCT comparing 8 months of aerobic versus resistance exercise in 155 overweight adults [21•], only aerobic exercise reduced HOMA and visceral fat. Similarly a RCT of obese postmenopausal women showed essentially no change in average insulin levels after 12 weeks of low-intensity resistance training [57].

#### Adipokines

Adipose tissue is an active endocrine organ, secreting bioactive factors known as adipokines [58] at abnormal levels in obesity. Some adipokines (TNF-α, IL-6) are mediators of a low-grade, systemic inflammatory state that is characteristic of obesity [59]. Adipokines, such as leptin, TNF-α, and IL-6, could mediate breast cancer development and progression directly by acting as mitogens in the breast, inhibiting apoptosis, and influencing tumor cell migration and invasion [15, 60]. They also may act indirectly, e.g., by enhancing estrogen bioactivity and promoting insulin resistance [15]. Conversely, adiponectin is an adipokine that occurs at lower levels in obesity and is anti-mitogenic, anti-inflammatory, and promotes insulin sensitivity. Epidemiologic findings relating adipokines in circulation to increased postmenopausal breast cancer risk are generally mixed, but suggestive, for increased leptin [61–63], decreased adiponectin [62–64], and a decreased adiponectin:leptin ratio [62]. There is weaker epidemiologic evidence of etiologic roles for IL-6 [65], TNF-α [63, 66], and the inflammatory marker C-reactive protein (CRP) [62, 65], produced in response to TNF-α and IL-6.

RCT findings overall imply that exercise *in conjunction with sufficient weight loss* can decrease circulating leptin [50 , 51, 67•] and perhaps IL-6 [43•, 68•] in postmenopausal women. Exercise-related decreases in CRP have been found in some RCTs of postmenopausal women [69–71], but not all [26], and in others, only with concurrent weight loss [68•, 72]. The Dose–response to Exercise in postmenopausal Women (DREW) study compared three doses of aerobic exercise (4, 8, or 12 kcal/kg/week at 50 % VO_2max_), but revealed no difference with respect to CRP change in exercisers versus controls over 6 months [72]. Six RCTs of postmenopausal women consistently showed no change in adiponectin levels, on average, with exercise alone [50, 53, 57, 67•, 69, 73]. Furthermore, in primary analyses from multiple RCTs there was no reported effect of exercise-only on TNF-α [53, 69, 70] or IL-6 [53, 68•, 69–71, 73] levels in postmenopausal women.

Weight loss is a plausible mediator of exercise-induced adipokine changes given strong biologic rationale and a growing body of RCT evidence, particularly for leptin. The NEW trial, for example, demonstrated up to a 53 % average decrease in leptin concentrations in subgroups with ≥10 % weight loss, which far exceeded changes in subgroups with <5 % weight loss (e.g., 5.5 % average decrease, exercise-only) [67•]. The mechanisms driving exercise-related inflammatory marker changes (CRP, TNF-α, IL-6) are probably more complex, relating not only to weight loss but potentially also to effects on muscle tissue, endothelial cells, and immune cells [59].

#### Immune function

Evading immunological destruction is an emerging hallmark of cancer and a diminished competence of the immune response is a recognized predictor of cancer risk; however, there is currently no consensus on which immune biomarkers are causally related to cancer risk [74, 75].

Physical activity might impede carcinogenesis by moderating the innate and adaptive immune systems [76] and, thus, enhance immunosurveillance and the tumor suppression capacity of the immune system. Furthermore, exercise could help to modulate obesity-related, proinflammatory immune responses [59] and prevent age-related immunosenescence [77]. To date, the acute, transient effects of exercise on immune function have been studied extensively, supporting beneficial effects with moderate-intensity but detrimental effects with high-intensity activity [76]. Although some studies have demonstrated altered number and function of circulating immune cells (e.g., enhanced natural killer cell cytotoxicity and T-lymphocyte proliferation capacity) with long-term exercise, there is a lack of supportive evidence from RCTs [76–78]. For example, the 12-month Physical Activity for Total Health study found no effect of aerobic exercise on *in vitro* natural killer cell cytotoxicity, T-lymphocyte proliferative response to stimulation, or the relative proportion of immune cell counts (e.g., T-cells, helper T-cells, cytotoxic T-cells, B cells, natural killer cells) [79].

#### Oxidative stress and telomere length

Oxidative stress results from an imbalance of increased systemic reactive oxygen species (ROS) production and/or reduced antioxidant capacity, including the ability to neutralize reactive intermediates and repair subsequent damage. It is hypothesized to play a central role in breast carcinogenesis [80] and in carcinogenic causal pathways linked to obesity [81]. ROS-induced damage to macromolecules leads to genetic alterations [82]. Because telomeres, nucleoprotein repeats at the ends of chromosomes that protect cells from chromosomal instability, suffer disproportionately from oxidative damage, an important etiologic pathway through which oxidative stress might affect breast cancer risk is through telomere attrition [83].

As part of a favourable biological adaptive response, regular exercise enhances antioxidant and oxidative damage repairing enzyme capacity and may subsequently reduce oxidative damage [84]. The epidemiologic evidence supporting that regular physical activity reduces oxidative damage to macromolecules or telomere attrition is, thus far, limited but suggestive. In the Physical Activity for Total Health study, there was decreased oxidative damage to lipids as measured by F_2_-isoprostane levels in exercisers compared to controls, with a more pronounced statistically significant reduction in exercisers who increased their physical fitness by >15 %; this effect occurred independent of obesity status [85]. RCTs of Tai Chi exercise [86] and resistance training [87] also showed reductions in oxidative damage. Yet with respect to telomere length, the NEW trial found no effect of dietary weight loss and/or aerobic exercise [88•], nor did a diet-physical activity lifestyle intervention RCT for diabetes prevention in high-risk adults [89].

#### Global DNA hypomethylation

The methylation of DNA is recognized as a key epigenetic mechanism in the regulation of gene expression and chromosomal stability. Global DNA hypomethylation in peripheral blood leukocytes represents a postulated biomarker for cancer risk [90] and epidemiologic evidence of an association with increased breast cancer risk is accumulating [91, 92]. Potential epigenetic modifications induced by exercise have been described [93•], and, to-date, two observational studies showed positive associations between physical activity and prevalent repetitive sequences (LINE-1) methylation, a surrogate measure of global methylation [94]. In middle-aged, white women with a family history of breast cancer, higher self-reported physical activity (≥9.8, 5.9, and 12.5 hours per week for childhood, teenage years, and past 12 months, respectively) was associated with a favorable 33 % increase of LINE-1 methylation [95]. Similarly, in another study, cancer-free adults with 26–30 minutes per day of recent physical activity (versus ≤10 minutes per day) as measured via accelerometer, had higher LINE-1 methylation [96].

#### 25-hydroxyvitamin D

A protective, inverse association between vitamin D exposure and postmenopausal breast cancer risk is becoming increasingly clear [97, 98]. For some individuals, outdoor physical activity could improve vitamin D status by increasing cutaneous production of vitamin D_3_ when UV-B exposure is sufficiently high. Another mechanism involves body composition, because the metabolite 25-hydroxyvitamin D (25(OH)D), the most common serum indicator of vitamin D status, might sequester in body fat [99]. Evidence from the NEW trial supports this hypothesis; in overweight/obese postmenopausal women, sufficient weight loss (≥15 % body weight) over 12 months, whether induced with aerobic exercise or caloric restriction, increased serum 25(OH)D concentrations significantly relative to controls (*p*-trend = 0.002) [100•]. Furthermore a 2-year weight loss trial of 383 overweight/obese women revealed a clear dose–response relation between increasing weight loss and increasing serum 25(OH)D concentrations (*p*-trend = 0.005), and in multivariable models, weight loss >10 % was identified as a significant predictor of 25(OH)D change [101].

## Proposed Biologic Model

### An updated model

**Fig. 1:**
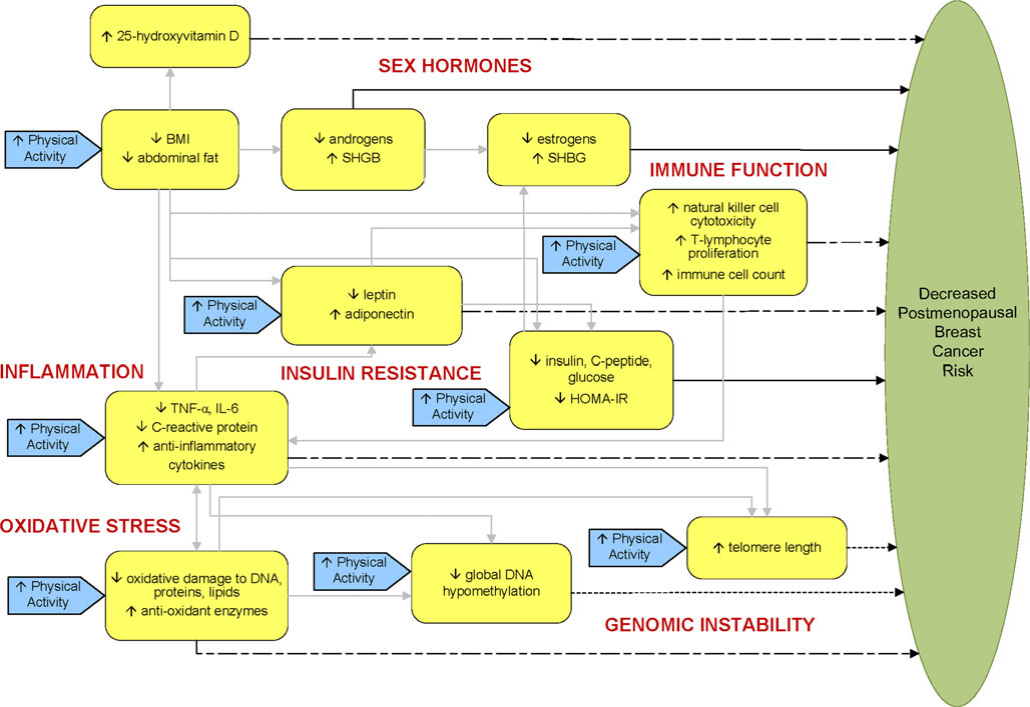
Hypothesized biological model relating physical activity to postmenopausal breast cancer risk. *Strong* epidemiologic evidence of an association with breast cancer risk (solid black arrows); *limited* epidemiologic evidence (irregular dashed arrows ); *emerging* epidemiologic evidence (short dashed arrows). Grey arrows relating biomarkers to each other are proposed in the literature; some of these relations are hypothesized, whereas others are well-established. Adapted from [11]

Figure 1 depicts a proposed, updated [8•, 11], biologic model relating physical activity to postmenopausal breast cancer risk via interrelated pathways with common linkages to adiposity. Notable exclusions from our model include mammographic density, which is a strong risk factor for breast cancer [102]; however, evidence from observational studies and RCTs overall do not support an association between long-term exercise and breast density [103]. Similarly, while elevated circulating IGF-1 might signify an increased risk for postmenopausal breast cancer [104], it has not been shown to be decreased with physical activity [50, 105, 106•]. Furthermore, we acknowledge that DNA damage, e.g., resulting from oxidative stress or genotoxic estrogen metabolites, could initiate breast cancer and that DNA repair mechanisms might be enhanced with physical activity [10]; however, this topic was beyond the scope of our review.

### Role of body fat

Adiposity change could play a mediating role for any of the biomarkers proposed in our model, or for some biomarkers, physical activity might act independently. The state of being overweight or obese also could modify the effects of physical activity on other biomarkers. Distinguishing the effects of “fat and fit” on breast cancer biomarkers is methodologically challenging and of interest [44, 54•, 107]. Four-armed randomized trials, such as the NEW trial and the recent SHAPE-2 study [108••], were designed to address the fit-versus-fat controversy directly and provide some of the clearest distinctions between exercise and weight loss. Published findings from the NEW trial and other RCTs in this review imply particularly that exercise-related changes in estrogens, SHBG, leptin, and 25-hydroxyvitamin D are mediated largely by weight loss in healthy postmenopausal women.

## Research Opportunities

While considerable advances have been made in our understanding of the biologic mechanisms relating physical activity to postmenopausal breast cancer risk, opportunity remains for future research. To begin, several newly hypothesized biologic pathways require further study to better understand their etiologic roles in postmenopausal breast cancer and hence, their suitability as biomarkers. Second, although our list of proposed biomarkers is fairly comprehensive, encompassing our own areas of research, additional biomarkers can be considered for our model, including, for example, those related to DNA repair mechanisms, additional epigenetic indicators, other adipokines, and anti-inflammatory cytokines. Several of these mechanisms may be more intensive to measure than what has been done previously, such as genomic alterations, and may require a tissue-specific approach. Hypothesized biomarkers also could be removed from the model as data becomes available; e.g., exercise-only trials in older women thus far have not produced changes in circulating adiponectin, TNF-α, or IL-6 levels. Third, biomarker-exercise RCTs could be analyzed using a systems epidemiology approach [109], quantifying the direct and indirect causal effects of exercise on multiple biomarkers simultaneously rather than on single biomarkers as in previous analyses. Results would identify pivotal exercise-induced biomarker changes and important mediators of those changes as targets for future intervention research. Fourth, new RCTs in healthy postmenopausal women are needed to compare different types of physical activity (e.g., aerobic versus resistance) and different doses (i.e., frequency, duration and intensity) to clarify the optimal prescription for breast cancer risk reduction. Studies exploring interindividual variability in exercise-induced biomarker changes, e.g., due to common genetic polymorphisms, would help tailor exercise prescriptions.

One recently completed, noteworthy study is the Breast Cancer and Exercise Trial in Alberta (BETA Trial). This trial, comprising 400 postmenopausal, previously inactive women who were randomized to undertake either 150 or 300 minutes per week of aerobic exercise for 1 year, was designed specifically to determine the optimal activity dose for lowering postmenopausal breast cancer risk. In secondary analyses of the ALPHA trial data, higher exercise duration was associated with desirable changes in adiposity and circulating sex steroid hormone levels, HOMA-IR, leptin, and CRP levels, with the greatest benefit observed for women undertaking >150 minutes per week [32] or >225 minutes per week [50, 70, 110] of aerobic exercise. Therefore, previous biomarker-exercise RCTs that generated null findings in primary analyses may have been limited by the relatively low dose of exercise that was prescribed or attained by study participants. Furthermore, it is possible that the current physical activity guidelines recommended for cancer prevention are insufficient for postmenopausal breast cancer. A recent prospective investigation of 30,797 postmenopausal women [111•] found no significant association between invasive breast cancer incidence and near-achievement of the WCRF/AICR 2007 minimum physical activity recommendations [1] for cancer prevention.

## Conclusions

Evidence from randomized exercise trials in healthy, overweight and obese postmenopausal women implies that moderate-vigorous aerobic exercise prescriptions of 150–225 minutes per week over 12 months can lower estradiol levels by approximately 5-10 % on average, primarily through total body weight loss. Yet, there is biologic plausibility that some exercise-induced biomarker changes do not require loss of body fat, whereas others depend on abdominal fat loss. The preventive effect of exercise is probably the culmination of numerous interrelated biomarker changes that, when combined, act additively or synergistically to impede carcinogenesis in the breast. The level of physical activity required to induce these changes could be higher than the minimum level currently advised for cancer prevention and might depend on individual factors, e.g., genetic constitution. Identifying a physical activity prescription that produces clinically meaningful changes in key biomarkers and subgroups of women who would benefit the most from physical activity is a priority for future research. These recommendations will be used to inform prevention strategies for breast cancer after menopause.
